# Gastroenteritis and Transmission of *Helicobacter pylori* Infection in Households^1^

**DOI:** 10.3201/eid1211.060086

**Published:** 2006-11

**Authors:** Sharon Perry, Maria de la Luz Sanchez, Shufang Yang, Thomas D. Haggerty, Philip Hurst, Guillermo Perez-Perez, Julie Parsonnet

**Affiliations:** * Stanford University School of Medicine, Stanford, California, USA;; † Santa Clara County Health and Hospital Systems, San Jose, California, USA;; ‡ New York University School of Medicine, New York, New York, USA

**Keywords:** Helicobacter pylori, gastroenteritis, children, incidence, serology, stool antigen, research

## Abstract

The mode of transmission of *Helicobacter pylori* infection is poorly characterized. In northern California, 2,752 household members were tested for *H. pylori* infection in serum or stool at a baseline visit and 3 months later. Among 1,752 person considered uninfected at baseline, 30 new infections (7 definite, 7 probable, and 16 possible) occurred, for an annual incidence of 7% overall and 21% in children <2 years of age. Exposure to an infected household member with gastroenteritis was associated with a 4.8-fold (95% confidence interval [CI] 1.4–17.1) increased risk for definite or probable new infection, with vomiting a greater risk factor (adjusted odds ratio [AOR] 6.3, CI 1.6–24.5) than diarrhea only (AOR 3.0, p = 0.65). Of probable or definite new infections, 75% were attributable to exposure to an infected person with gastroenteritis. Exposure to an *H. pylori*–infected person with gastroenteritis, particularly vomiting, markedly increased risk for new infection.

## Abstract 2

In northern California homes, exposure to gastroenteritis in an *H. pylori–*infected contact markedly increased *H. pylori* infection.

Helicobacter pylori infects at least 50% of the world's population. Infection occurs in early life (*1**,**2*). Because acute infection invariably passes undetected, however, the precise age of acquisition is unknown. In industrialized countries, infection rates are declining rapidly (*1**,**3*), but high rates of infection persist among disadvantaged and immigrant populations (*4**,**5*).

The mechanisms of H. pylori transmission are incompletely characterized. Person-to-person transmission is most commonly implicated with fecal/oral, oral/oral, or gastric/oral pathways (*6*); each has supportive biologic as well as epidemiologic evidence. Like many common gastrointestinal infections, infection is associated with conditions of crowding and poor hygiene (*7**,**8*) and with intrafamilial clustering (*9**–**12*). The organism has been recovered most reliably from vomitus and from stools during rapid gastrointestinal transit (*13*). These findings raise the hypothesis that gastroenteritis episodes provide the opportunity for H. pylori transmission.

Household transmission of gastroenteritis is common in the United States, particularly in homes with small children (*14*). If H. pylori is transmitted person to person, one might expect rates of new infection to be elevated after exposure to persons with H. pylori–infected cases of gastroenteritis. To explore whether diarrhea or vomiting contributes to disease transmission, we monitored northern Californian households experiencing gastroenteritis for new H. pylori infections and evaluated the contributions of household H. pylori infection and gastroenteritis to new infection. We were also interested in whether symptoms of new infection could be identified.

## Methods

### Population and Study Design

The study population consisted of households that were participating in the Stanford Infection and Family Transmission study, initiated in 1999 to prospectively evaluate the association of H. pylori infection and household gastroenteritis (*14**,**15*). The cohort, predominately Hispanic immigrant families residing in South San Francisco Bay, has a high seroprevalence of H. pylori infection (*16**,**17*). From January 2000 to July 2004, a total of 3,846 persons seen at 1 of 15 community (predominately low-income public health) clinics with diarrhea, vomiting, or both, were asked for permission to be contacted by a study representative for a phone screening. Of 2,941 households that could be contacted, 2,155 (73%) were eligible multiperson households (within 50 miles of the research site, within 12 days of gastroenteritis onset of index patients). Of these, 852 (40%) declined interest, and 1,303 (60%) were scheduled for a home visit. At the first home visit, consenting household members were interviewed regarding symptoms, onset, and duration of gastroenteritis within the previous 21 days. Blood and stool samples were collected for H. pylori testing.

Stool samples were requested from children <2 years of age and from others who refused phlebotomy. Approximately 3 months later (range 12–20 weeks), household members were reinterviewed, and a second specimen was collected for H. pylori testing. Participation was voluntary; small gifts (i.e., a mug, a tote bag, hand antiseptic, a calendar) were offered as thanks for participation. The study was approved by the institutional review boards of Stanford University, Santa Clara and San Mateo Counties, and the state of California.

### Laboratory Methods

#### **H. pylori** Serologic Testing

Anti–H.pylori immunoglobulin G (IgG) was quantified by using an in-house ELISA (*18*), previously validated and adapted for use in US, Latin American, and Asian populations (*19**–**21*). Optical density (OD) results were categorized as negative (<85% of low positive standard), borderline (85%–110% of low positive standard), or positive (>110% of low positive standard). In different series of control samples with biopsy-confirmed infection, the assay, which includes 6 different isolates (2 from Mexico), is 91% sensitive and 98% specific for infection in adults (*15**,**18*). For our study, an equivalent sensitivity and specificity were established for children >2 years of age by using samples from controls with biopsy-confirmed infection from a separate study to establish a lower OD cut-off (75% of adult control) (*15*). Serologic testing results are considered unreliable in children <2 years of age (*22*).

Each serum sample was tested in triplicate for H. pylori on 2 occasions, soon after it was received and later, when it was paired with the second visit sample from the same study participant. Between testing, samples were frozen at –80°C. The paired serum results are presented here. High reproducibility between first and second runs of the same sample (mean coefficient of variation 18 ± 17) suggests that the effect of freezing or storage was negligible. Titer levels were derived from ODs by standard curve methods. A seroconversion was defined as a qualitative change from negative to positive, negative to borderline, or borderline to positive, if accompanied by >4-fold increase in H. pylori titer from baseline. A seroreversion was defined as a qualitative change from positive to negative, accompanied by >2-fold decrease in H. pylori titer.

To corroborate recent H. pylori infection (*23*), serum samples from 22 IgG seroconverters and 22 randomly sampled, persistently seronegative adults were tested for H. pylori IgM antibody response in the laboratory of Dr. Perez-Perez by using a mixed strain assay previously validated in ethnically diverse and pediatric populations (*9**,**24*). Detection of an IgM antibody response at either first or 12-week follow-up visit was considered a positive test result.

#### **H. pylori** Stool Antigen

Stool antigen was tested with the Premier Platinum HpSA enzyme immunoassay (Meridian, Cincinnati, OH, USA). Stool samples collected at home visits were transported to the laboratory and stored at –20°C until processed. Samples not available at the home visit were expressed by overnight mail. In 1 metaanalysis, stool antigen had a mean sensitivity and specificity of 91% and 93%, respectively (*25*); however, accuracy may be lower in children <6 years of age (*26*). In the present study population, H. pylori was identified by PCR in 12 (46%) of 26 transiently positive stool samples from toddlers (vs. 0% of 10 stool antigen–negative samples), a finding that was consistent with the 50% sensitivity of PCR observed in H. pylori–inoculated stools (*27*). A stool conversion was defined as a qualitative change from negative to positive when the manufacturer's suggested cut-off values were used.

#### Testing Protocol

We requested stool and serum from all participants >2 years of age, although participants were included in the study if they offered 1 or the other. For practical reasons (i.e., stool samples are not typically available on demand and are unpleasant to ship) most persons >2 years of age (97%) provided only blood samples. Approximately 29% of children <2 years of age provided only blood samples, and 39% provided both blood and stool samples. The ^13^C urea breath test was not considered because of costs and the accuracy of the applied methods (*22*).

## Definitions

### Gastroenteritis

Gastroenteritis was defined as either 1) >3 loose or watery stools per day in persons at least 2 years of age, or >5 per day in children <2, or 2) any vomiting. Symptoms lasting >14 days or reported symptoms due to potentially noninfectious causes (poisoning, pregnancy, chemotherapy) were excluded from the case definition (*14**,**15*). Gastroenteritis was categorized as vomiting with or without diarrhea, or diarrhea alone. Only cases of gastroenteritis identified at the baseline visit were included in the present analysis.

### New Infection

Given low expected rates of new infection and the role of testing error (28,29), we developed criteria for definite, probable, and possible infection on the basis of corroborative test results. A definite new infection was a seroconversion with confirmatory stool or IgM result. A probable new infection was a borderline seroconversion with confirmatory stool or IgM or a stool conversion with confirmatory IgG or IgM. Possible new infections met criteria for serologic or stool conversion without confirmation. Since serum specimens were not routinely obtained from children <2 years of age, a single stool conversion was the only way of diagnosing new infection in most of these children.

### Persistent Negative/Persistent Positive

A persistent negative infection had no positive or equivocal test results during observation. A persistent positive infection had no negative or equivocal test results during observation. A transient infection was defined as a qualitative seroreversion with >2-fold decrease in H. pylori titer or a qualitative (positive to negative) stool reversion. Study participants with equivocal (40 qualitative seroconversions with <4-fold increase in H. pylori titer and 32 qualitative seroreversions with <2-fold decrease in H. pylori titer) or transient (7 serology and 28 stool) test results were not considered for outcomes but were included as baseline household exposures.

### Household Exposure

A person was considered exposed to gastroenteritis, H. pylori infection, or H. pylori infection with gastroenteritis if >1 other person in the home had 1 of these profiles at the baseline visit. Household members who were not available for second visit testing (499 or 15% of baseline participants) were counted as baseline exposures but could not be considered for outcomes.

### Statistical Analysis

#### Incidence of New Infection

We estimated the annual incidence of new infection and 95% confidence intervals (CIs) (*30*) for all ages and for age categories 0–2, 2–17, and >18 years. The outcome was the proportion of new infections in the age category, and the denominator was the total number of study participants in the age category who tested negative at baseline and who completed the second visit testing. For comparison, annualized rates of seroconversion and seroreversion are also reported. The denominator for estimating seroreversions included all persons who tested positive at the first visit and who completed second-visit testing.

#### Classification and Symptoms of New Infections

Classification of new infections (definite, probable, or possible) is described. The χ^2^ test or Wilcoxon test for categorical and continuous measures, respectively, and logistic regression for multivariable adjustment were used to evaluate symptoms and other characteristics of new infections versus persistent negative results.

#### Incidence of New Infection by Household Exposure

We compared rates of new infection for study participants who resided with no known H. pylori–infected contact, with >1 infected contact, and with >1 infected contact who had gastroenteritis. The denominator for these estimates was the number of study participants within each exposure category who tested negative at baseline and who completed second-visit testing. Household members who were not available for second-visit testing or who completed both visits with equivocal changes in titer were counted as baseline exposures but could not be considered for outcomes.

#### Exposure to Gastroenteritis in an **H. pylori**–Infected Contact

Among study participants residing with >1 H. pylori–infected contact, we estimated the odds (and 95% CIs) of new infection, given exposure to infection with versus without gastroenteritis, including symptoms of gastroenteritis (vomiting versus diarrhea only). To minimize misclassification, the outcome for this analysis was restricted to definite and probable new infections, and the reference group with persistent negative results who had been exposed to H. pylori infection at baseline. A random intercept logistic regression model (Proc Glimmix., SAS/Stat, 9.2 ed., SAS Institute, Cary, NC, USA) was used to model household clusters and to adjust for age, sleeping density, and proportion of household members completing both visits, each modeled as a continuous variable. We also calculated the attributable risk (*30*) of new infection associated with exposure to H. pylori infection and to exposure to H. pylori infection with gastroenteritis. As a secondary analysis, we assessed risk factors for all new infections, including possible new infections.

## Results

From January 2000 to June 2004, a total of 1,186 households were enrolled. These 1,186 households included 6,620 participants who participated in the first gastroenteritis interview, and 4,334 (65%) who also gave specimens. Households were predominately Spanish-speaking and of low income (Table 1). Nearly three quarters (72%) of households had >1 H. pylori–infected household participant at the first visit.

**Table 1:** Household characteristics

Characteristics	Enrolled households (n = 1,186)	Households completing follow-up (n = 909)	Households not completing follow-up (n = 277*)*	p value
No. contacts, median (range)	6 (2–21)	5 (2–21)	6 (2–19)	0.06
No. enrolled, median (range)	3 (2–17)	3 (2–17)	3 (2–10)	0.03
No. children enrolled, median no. <18 y (range)	2 (0–11)	2 (0–11)	2 (0–9)	0.95
Educational attainment, median highest year (range)	12	12	12	0.42
% Spanish-speaking	72	73	71	0.65
Sleeping density (median persons/bedroom)	2.3/bedroom	2.5/bedroom	2.3/bedroom	0.66
Income (%<US$30,000/y)	59	58	62	0.39
>1 *Helicobacter pylori* infected (%)	72	72	70	0.42
Emergency dept. referral (%)	16	15	21	0.02

Of enrolled households, 277 (23%), including 108 that dropped out and 169 that could not be located, did not complete the second visit (Table 1). Although the 909 households that did complete the study appeared somewhat smaller (median 5 vs. 6 household members, p = 0.06 Wilcoxon), the proportion of large households (>8 contacts) was not significantly different (20% vs. 19%). Conversely, enrollments (median 3 per household) were similar, although completing households were somewhat more likely to enroll more than the minimum of 2 participants (72% vs. 64%). Households that did not complete the study were also more likely to have been referred through an emergency department (21% vs. 15%, p = 0.02) but were similar in number of children enrolled, primary household language, sleeping density, educational attainment, and prevalence of H. pylori infection.

### Incidence of New Infection

The 909 households that completed the second visit had 3,380 household participants; 2,881 (85%) completed both specimen collections. Of these, 129 (4.4%) were children <2 years of age who contributed only serum and were excluded from analysis because of lack of validation of our ELISA results in early childhood. Thus, a total of 2,752 household members, 2,372 (86%) with serologic results and 380 (14%) with stool or stool and serologic results, completed second-visit testing. Of these, 1,752 (64%) tested negative at baseline. Over a median 13 (±2) weeks of follow-up, 30 (1.7%) of these met the definition of a new infection, for an overall annualized incidence of 6.8% (95% CI 4.6%–9.8%). By serology, corresponding annualized seroconversion and seroreversion rates were 3.7% and 3.1%, respectively. Half of all new infections occurred in children <2 years of age, for an annualized rate of 20.9% (95% CI 11.8%–33.9%) in this age group versus 5.3% (2.4%–9.6%) in persons 2–17 years, and 3% (0.9%–6.5%) in persons >18 years.

### Classification and Symptoms of New Infections

Among the 30 new infections, 7 met criteria for a definite new infection, 7 for a probable new infection, and 16 for a possible new infection (Table 2). The 7 definite IgG seroconversions included 4 corroborated with IgM and 3 seroconversions corroborated with change in stool antigen. Of the 7 probable new infections, 2 stool conversions (both in children <2 years of age) were corroborated with IgM, 4 borderline seroconversions were corroborated with IgM, and 1 borderline seroconversion was corroborated with stool antigen. Overall, 10 (45%) of the 22 stool or seroconversions tested had a positive IgM response at the first or second visit of the study participant, compared to 4 (18%) of 22 randomly selected persistent seronegatives (p = 0.05). The 16 possible new infections included 12 stool conversions, all in children <2 years, and 4 uncorroborated borderline seroconversions.

**Table 2:** Case listing of 30 new infections*

Household–contact no.	Age (y)	GE	Criteria			No. *Helicobacter pylori*–infected contacts		
			IgG	Stool	IgM	Without GE	With diarrhea	With vomiting
Definite								
1–1	1.2	D/V	+	+	-	1	0	0
2–1	1.3	D/V	+	+	-	2	0	0
3–1	1.5	D/V	+	+	-	2	1	0
4–3	10	D/V	+		+	0	0	2
5–5†	11	D/V	+		+	0	3	1
6–3	23	D	+		+	0	0	1
7–2	37	D	+		+	3	0	1
Probable								
8–1	0.74	D/V		+	+	1	0	0
9–1	0.77	V		+	+	0	0	0
10–1	2.3	D/V	+ (B)	+		0	0	0
11–1	3.6	D/V	+ (B)		+	0	1	0
12–2†	21		+ (B)		+	2	0	0
13–2	23	D	+ (B)		+	0	0	1
14–2	42		+ (B)		+	0	0	0
Possible								
15–5	0.3			+		0	0	0
16–1	0.33	D		+	-	1	0	0
17–1	0.6	D/V		+	-	4	0	0
18–1	0.8	D/V		+		0	0	0
19–1	0.9	D/V		+	-	1	0	0
20–1	1.0	D		+	-	1	0	0
21–1	1.1	D/V		+	-	1	0	0
22–1	1.2	D/V		+		0	1	0
23–1	1.3	D/V		+	-	0	0	0
24–1	1.9	D/V		+		3	0	0
25–1	6.3	D		+		0	0	0
26–3	7		+ (B)		-	1	0	1
5–3†	7.6	D/V	+ (B)		-	0	3	1
28–1	7.8	V	+ (B)		-	3	0	0
29–4	8.5			+		2	0	0
12–4†	12.2		+ (B)		-	0	0	0

*GE, gastroenteritis; D, diarrhea only; V, vomiting only; D/V, diarrhea with vomiting; B, borderline seroconversion with >4-fold increase *in H. pylori* titer. †Cases occurring within the same household.

Overall, 175 children <2 years had serologic and stool results for each visit. Among 350 stool serum pairs, 335 (96%) were concordant, including 3 of 10 new infections and 2 transient infections. Of the 15 discordant results, 7 were stool conversions discordant at the study participant's second visit, 6 were stool reversions discordant at the participant's first visit (4 of these were corroborated by PCR in another study [27], and 2 were persistently stool positive or negative with discordant serology at 1 or both study participant visits).

Compared with 1,722 persistently negative results, the 30 new infections were in significantly younger study participants (median age 2 vs. 11 years, p<0.001) but of similar gender (40% male vs. 43% male, p = 0.78). When results were adjusted for age, new infections were somewhat more likely than persistently negative results to be in persons with gastroenteritis (adjusted odds ratio [AOR] 2.5, CI 0.97–6.6, p = 0.06), and the 14 definite or probable new infections were nearly 5 times more likely (AOR 4.9, CI 1.1–22.4, p = 0.04). No specific gastroenteritis symptoms for new infection were identifiable.

### Incidence of New Infection by Household Exposure

Seven (*7*) new infections occurred in homes with no known H. pylori–infected participants (Figure) and 23 (77%) in homes with >1 infected contact, for rates of new infection of 1.1% and 2.1%, respectively (p = 0.10). Two households with exposure to an H. pylori–infected contact manifested 2 new infections (Table 2). Conversely, 1,319 (75%) study participants were exposed to gastroenteritis in another household member; new infection developed in 16 (1.2%), compared with 14 (3.2%) of 433 study participants not exposed (p = 0.005). However, new infection developed in 10 (2.9%) of 350 study participants exposed to an H. pylori–infected contact with gastroenteritis, compared with 6 (0.6%) of 969 of study participants exposed to gastroenteritis in an uninfected contact (p = 0.001).

**Figure:**
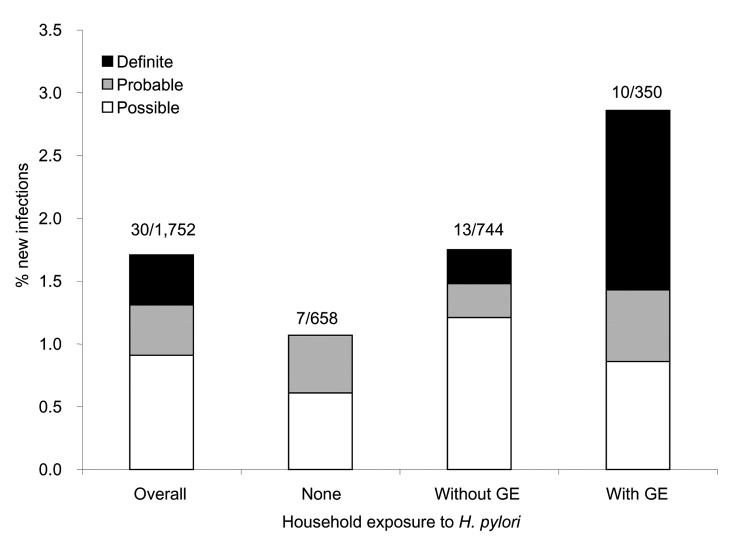
Rates of new *Helicobacter pylori* infection overall, without exposure to an infected contact (none); to >1 infected contact without gastroenteritis (Without GE), or to >1 infected contact who had gastroenteritis (With GE). Bar annotations denote number of new infections and number at risk. Definite/probable/possible, see text for classification of new infections.

### Exposure to Gastroenteritis in an H. pylori–Infected Contact

Of 14 definite or probable new infections, 11 (79%) occurred in homes with >1 H. pylori–infected contact (Table 2). When adjusted for age, sleeping density, and proportion of household contacts completing both visits, exposure to an H. pylori–infected person with gastroenteritis increased risk for definite or probable new infection 4.8-fold (95% CI 1.4–17.1, p = 0.01) compared with exposure to infected persons without gastroenteritis. Exposure to an H. pylori–infected person with vomiting was a significantly stronger risk factor for new infection (p = 0.03) than exposure to an H. pylori–infected person with gastroenteritis but no vomiting (Table 3). The proportions of definite or probable new infections attributable to exposure to an H. pylori–infected person without and with gastroenteritis were 55% and 75%, respectively. Including possible new infections in the analysis decreased the magnitude of the associations with gastroenteritis (Table 3) although exposure to an H. pylori–infected person with vomiting remained a significant risk factor.

**Table 3:** Risk factors for new infection in households with >1 *Helicobacter–pylori* infected participant*

Symptoms of H. pylori–infected household contact	Definite/probable new infections *(*n = 14 in 555 households)		All new infections (n = 23 in 566 households)	
	AOR	95% CI	AOR	95% CI
No GE	1.0		1.0	
GE w/ vomiting ± diarrhea	6.3	1.6–24.5	2.9	1.0–8.1
GE w/ diarrhea only	3.0	0.5–17.2	1.6	0.4–6.2

*AOR, adjusted odds ratio (random intercept model (household), adjusting for age, sleeping density, proportion of household completing both visits); CI, confidence interval; GE, gastroenteritis.

## Discussion

In this prospective study of H. pylori infection and household gastroenteritis within a US immigrant population, we estimated an annualized H. pylori incidence rate of 7%, including 21% among children <2 years of age. Exposure to H. pylori–infected persons with gastroenteritis, particularly with vomiting, increased risk for new infection, and three quarters of definite or probable new infections were attributable to exposure to H. pylori infection with gastroenteritis. These findings indicate that in US immigrant homes, H. pylori transmission occurs in young children during household episodes of gastroenteritis.

Exposure to an infected household member with vomiting was associated with a 6-fold greater risk for new infection, whereas exposure to diarrhea elevated, but not significantly, the risk for new infection. These findings are consistent with prior research that shows that H. pylori is recovered reliably from vomitus (up to 30,000 CFU/mL) and can also can be grown from aerosolized vomitus collected at short distances (<1.2 m) (*13*). Epidemiologic investigations also implicate vomitus as an effective vehicle for gastro-oral transmission (*31**,**32*). Although found in diarrheal stools (*13*), H. pylori is not reliably grown from normal stools (*33*). The association between H. pylori and gastroenteritis is thus similar to that of other enteric pathogens that can be transmitted by vomitus or aerosolized vomitus or by the fecal-oral route (*34**,**35*). Although we cannot exclude other mechanisms of transmission in these homes, exposure to vomitus in an infected contact explained >50% of all new infections and >70% of definite and probable new infections.

Among our interests was identifying symptoms of new H. pylori infection. In experimental exposure, acute infection causes mild to moderate epigastric discomfort or dyspepsia in most study participants within 2 weeks, but symptoms are unlikely to be clinically detected (*36*). Although H. pylori–specific IgM antibodies may appear within 4 weeks, the frequency of this response is variable, particularly in children and when, as here, the time of infection is unknown (*37*). We did not observe a pronounced difference in the frequency or distribution of symptoms associated with new infection, although vomiting tended to be more frequent among persons with definite or probable new infections. Although the relatively small numbers of new cases may have limited the power of this analysis, no symptom complex was identified that would permit differentiation of acute H. pylori infection from other enteric processes. Because we did not establish the specific etiologic agent of gastroenteritis episodes, further studies are needed to more fully address this question.

Half of new infections were in children <2 years of age, and 2 of 3 were identified by a single unconfirmed stool conversion. Although H. pylori infection is acquired in early childhood, age of acquisition has been difficult to establish because of known limitations of existing noninvasive tests in very young children. In a Bogalusa, Louisiana, birth cohort, for example, the highest seroconversion rate (2%) was seen in children 4–5 years of age (*2*). Although stool antigen and urea breath tests are considered more accurate (*22*), studies in very young children are still limited. When the urea breath test was used, an annualized conversion rate more similar to ours (20%) was observed in the US–Mexican binational Pasitos cohort of children followed up from birth to 2 years (*38*). If, as suggested by this and other studies (*24**,**27**,**39**,**40*), acquisition with transient infection in early life often precedes persistent infection, rates of acquisition might be elevated when exposure to gastroenteritis is frequent. While the extent to which testing error confounds pediatric incidence studies is not clear, rates of acquisition among children in homes at high risk may nonetheless be meaningful measures of transmission risk.

Given the possibility of error in serologic and stool antigen tests, we cannot exclude the possibility that 30 new infections would occur by chance (*28**,**29*). Among those tested by serology, for example, the reversion rate was roughly equivalent to the seroconversion rate, which suggests that false-positive tests were occurring equally in the first and second screening. Because the predictive value of a single test in a low prevalence population may be <50%, we used a corroborative testing algorithm and restricted our risk factor analysis to those who had a confirmatory test of conversion. Despite the small number of cases, an association with exposure to H. pylori and gastroenteritis was highly significant. Although CIs were wide, the fact that risk estimates were uniformly strengthened in the subset of persons with corroborative test results lends validity to this approach, as well as underscoring the desirability of using a second test more routinely in incidence studies.

Although households that completed the study were largely representative of enrolled households, a substantial proportion of contacts in homes that completed the study either declined to participate (35%) or did not complete the second visit (15%). While rates of baseline H. pylori infection were virtually identical in those completing 1 or 2 visits, and participation rates were largely similar across household exposure profiles, we cannot exclude the possibility that the missing data were meaningful. If misclassification of gastroenteritis did occur, linkage with outcome is unlikely, since H. pylori infection status is not typically known. For these reasons, we assume that misclassification was random, minimizing the magnitude of true associations.

In summary, this study corroborates the conclusion that gastroenteritis, particularly with vomiting, in an H. pylori–infected person is a primary cause of transmission of H. pylori in humans. Despite some study limitations, the strength of association observed suggests an important milieu for future work to elucidate transmission pathways in low prevalence countries. As with other enteric infections such as hepatitis A, shigellosis, and cholera, H. pylori infection rates have decreased dramatically with improvements in sanitary infrastructure and household hygienic practices. Despite these trends, acquisition and infection are likely to remain prevalent in households with preexisting H. pylori infection, crowded living conditions, and frequent gastroenteritis.
